# Exploring Atopic Dermatitis in Indian Children: A Comprehensive Survey

**DOI:** 10.7759/cureus.89048

**Published:** 2025-07-30

**Authors:** Sneha Menon, Ashita Bhatia

**Affiliations:** 1 Medical and Safety Sciences, Research and Development, JNTL Consumer Health (India) Private Limited, Mumbai, IND; 2 Medical and Safety Sciences, Research and Development, Johnson &amp; Johnson Private Limited, Singapore, SGP

**Keywords:** atopic dermatitis, children, early intervention, moisturizers, sensitive skin

## Abstract

Introduction: Atopic dermatitis (AD) is a prevalent pediatric sensitive skin condition, yet data on parental awareness and preventive practices in India remain limited. This study aimed to assess parents' knowledge, attitudes, and practices (KAP) regarding AD in children under five years of age.

Methods: A cross-sectional, nationwide online survey was conducted among 1,003 Indian parents, selected from a pool of 3,012 respondents to ensure regional representation. Administered by Censuswide, the survey adhered to the British Polling Council, the Market Research Society (MRS), and the European Society for Opinion and Marketing Research (ESOMAR) ethical guidelines. The 20-item questionnaire primarily employed a Likert scale to assess parental knowledge, management strategies, and attitudes toward AD. Descriptive statistical analyses (frequencies, means, and percentages) were applied, with data stratification by demographics to enhance result interpretation.

Results: A survey conducted among 1,003 Indian parents of children under five years of age reported an AD prevalence of 17.85% (179 cases). Among the respondents, 687 (68.49%) were aware of strategies to delay AD onset in high-risk children, and 895 (89%) expressed interest in learning about preventive measures. Of the 179 children diagnosed with AD, 144 parents (80.45%) reported attempting preventive treatments prior to the diagnosis. The primary barrier to initiating preventive measures was a lack of awareness. Approximately 575 (57.3%) parents indicated a willingness to use moisturizers proactively before symptom onset in high-risk children. However, only 130 parents (12.96%) specifically identified moisturizers as their preferred preventive strategy for children at risk of developing AD. Parents prioritized safety, efficacy, and dermatologist recommendations when selecting topical treatments.

Conclusion: The findings highlight substantial parental interest in AD prevention, although gaps in awareness remain a critical barrier. These insights emphasize the need for targeted educational initiatives and early intervention strategies to enhance AD management and prevention in India. Further research utilizing inferential statistical analyses could provide deeper insight into the factors influencing parental decision-making and intervention effectiveness.

## Introduction

Pediatric sensitive skin is commonly linked to conditions like atopic dermatitis (AD), allergies, and other inflammatory skin disorders. These may manifest as burning, itching, tingling, dryness, redness, or skin thickening [1,2]. The spectrum includes eczema, contact dermatitis, xerosis, psoriasis, and rosacea-like dermatitis [3]. A compromised skin barrier plays a pivotal role in AD development among children with sensitive skin [4]. Additionally, genetic predisposition, environmental pollutants, and unhealthy lifestyles can further exacerbate AD [5].

AD's prevalence ranges from 15% to 23% in children [5]. Family history is a significant risk factor for AD [6], while sensitive skin is associated with immune-inflammatory processes, increased vascular reactivity, impaired skin barriers, and sensory nerve dysfunction [7]. One key factor underlying the heightened prevalence of AD in Indian children is the disruption of the skin's protective barrier. Genetic variations, such as defects in the FLG gene encoding filaggrin, can compromise the skin's structural integrity, making it more susceptible to environmental irritants and allergens [8]. This barrier dysfunction not only increases the risk of sensitization but also facilitates the penetration of allergens, triggering an exaggerated immune response and perpetuating the inflammatory cycle characteristic of AD. Early life environmental exposures significantly influence atopic disease development. AD typically presents more diffusely in infancy, and as the child grows, lesions become more localized to skin folds and joint creases [9]. Diagnosis is primarily clinical but may require allergological evaluation in certain cases [10]. However, differential diagnosis is crucial due to the clinical overlap with other dermatological conditions [11].

Early intervention is pivotal in managing AD in children and preventing the progression of the atopic march [12]. Early management should include topical moisturizers, anti-inflammatory medications (depending on the severity of the condition), and allergen/irritant avoidance [10]. Despite advancements, managing AD remains complex due to its multifaceted nature. The primary goal is to enhance quality of life by minimizing flare-ups and maintaining long-term disease control, considering factors such as geographical location, economic status, and genetic variations [13]. According to a British study, educating parents on how to apply moisturizers correctly resulted in an 800% increase in the number of moisturizers used, a drop in eczema severity, and a decrease in the proportion of patients requiring strong or moderate topical steroids. Physicians should assist parents of infants with infantile eczema in comprehending the value and necessity of using moisturizers to treat and prevent flare-ups of the condition [14].

The management of AD in the pediatric population in India is hindered by several factors. Key barriers include limited awareness and misconceptions about AD [15], along with insufficient knowledge of available treatment options and their efficacy [16]. Additionally, restricted access to healthcare and a lack of comprehensive data on the disease burden further hinder effective management [17]. Also, the burden of disease and unmet needs in the diagnosis and management of AD in India are not well-documented [18]. Improving the patient-clinician and parent-clinician partnerships in AD management is essential, as parents often lack sufficient information about the disease and its treatment [19].

Study objectives

Primary Objective

The primary objective was to assess parental knowledge, attitudes, and practices (KAP) regarding AD in Indian children under five years of age.

Secondary Objectives

The secondary objectives of the study were as follows: to evaluate parental awareness about early signs, symptoms, and preventive measures related to AD; to assess parental willingness to adopt preventive and treatment strategies, particularly regarding moisturizer use; and to identify gaps in parental knowledge and barriers to effective AD management.

The findings will provide valuable insights for healthcare providers, policymakers, and educators, highlighting the needs and challenges faced by families managing AD. These insights will support the development of targeted educational programs and medical interventions to improve AD management in India.

## Materials and methods

The present survey assessed parents' knowledge about AD, their attitudes toward the condition, and their practices in managing their children's symptoms.

Study design and participants

This study employed a cross-sectional survey design to investigate the knowledge, attitudes, and practices of parents regarding AD in children. The survey targeted parents across India. It was conducted online and executed by Censuswide, a research organization adhering to the British Polling Council, Market Research Society (MRS) code of conduct, and the European Society for Opinion and Marketing Research (ESOMAR) principles, ensuring ethical standards that prioritize participant welfare, client requirements, and community implications. Participants were enrolled through an existing online access panel managed by Censuswide. This panel consists of double opt-in members, requiring both an initial opt-in and a subsequent validation process. Members complete a profiling questionnaire upon joining, enabling targeted selection based on specific demographics and sectors. From a total pool of 3,012 parents, 1,003 Indian parents were selected based on their alignment with the study criteria. The respondents were a representative sample from across India, including the northern, southern, eastern, and western regions. While detailed urban/rural stratification was not collected, the sampling strategy was designed to ensure broad national coverage. Inclusion criteria included being a parent residing in India with at least one child under five years of age. No explicit exclusion criteria were applied beyond these parameters. The survey recruitment was incentivized with rewards varying from points redeemable for prizes to cash and air miles, depending on the survey's length.

Study instrument and data collection

The survey was conducted from 9 June to 12 June 2023, with participants invited via email. The questionnaire consisted of 15 main items with multiple subsections, totaling 20 questions in English (Appendix A). A Likert scale was primarily used to assess the severity, management, and impact of AD on children's daily lives. Additionally, the survey included a specific consent question at the beginning, allowing participants to opt in actively. Participants could withdraw at any stage by selecting “No” to the consent question or by simply closing their browser window, which ensured voluntary participation, implied consent, and respect for respondent autonomy.

Ethical considerations

The study's ethical approach was framed by Censuswide's commitment to the MRS code of conduct and ESOMAR principles, ensuring the welfare of participants. All personal data collected during the survey were stored securely, used solely for research purposes, and handled in compliance with relevant data protection regulations to maintain confidentiality and participant privacy.

Statistical analysis

The collected data were analyzed to determine the prevalence and characteristics of AD among the pediatric population in India. Descriptive statistics were used to summarize demographic and clinical characteristics. Results were reported as means, frequencies, and percentages, as appropriate. The responses were collated and stratified by demographics. 

## Results

This study included 1,003 parents of children under five years of age, representing diverse regions across India. The geographic distribution comprised 257 participants from North India, 442 from South India, 145 from West India, 106 from East India, and 53 from Northeast India.

Occurrence of AD in children

Overall, 179 (17.85%) participants had children with AD. The survey revealed that 111 (19.93%) male children and 68 (15.25%) female children had AD. Notably, the prevalence of AD was highest among three-year-old children, accounting for 44 (20.66%) of the participants. In terms of regional distribution, 11 (20.75%) of children from Northeast India were affected by AD.

Risk factors for AD

The present survey found that among 1,003 parents, 655 (65.30%) reported a family history of AD, as detailed in Table 1.

**Table 1:** Presence of family history of atopic dermatitis among 1,003 parents

Response	N (%)
Yes	655 (65.30)
No	(34.70)

A higher proportion of parents with male children (383; 68.76%) reported a family history of AD compared to those with female children (272; 60.99%). Regionally, this trend was most prominent in Western India, where 101 respondents (69.66%) indicated a family history of AD. Notably, 505 parents (50.35%) believed that a family history of food allergies could increase the likelihood of their child developing AD. It is important to note that this reflects parental perception rather than a validated clinical risk. Similarly, 543 parents (54.14%) identified with food allergies, and 422 (42.07%) identified hay fever as potential risk factors for AD, further highlighting common beliefs regarding familial and allergic predispositions.

Awareness of delaying AD symptom onset in high-risk children

**Figure 1:**
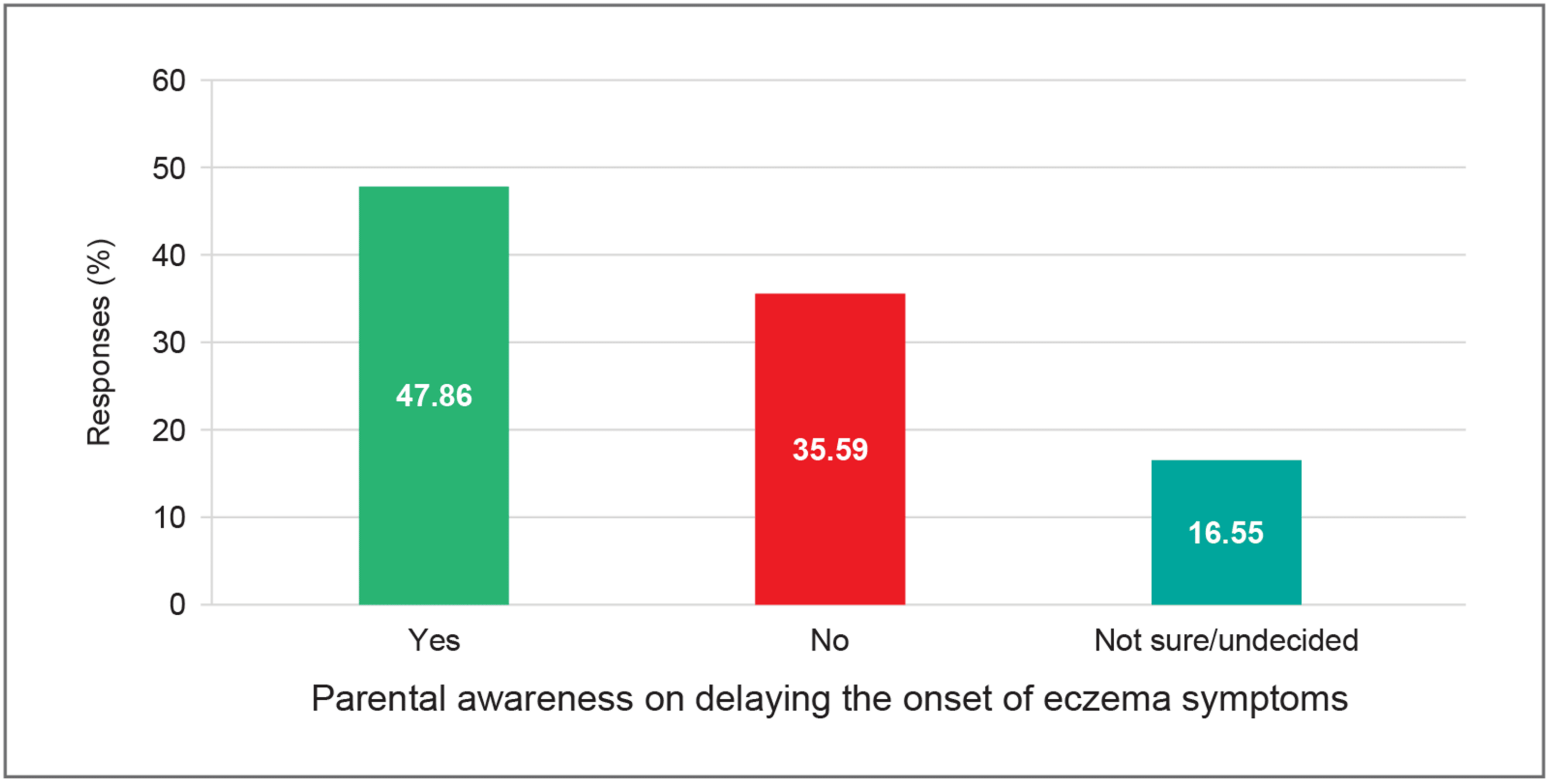
Parental awareness of delaying the onset of eczema symptoms

Among the 1,003 parents surveyed, 684 (68.4%) demonstrated awareness of strategies to delay the onset of AD symptoms in high-risk children, whereas 167 (17%) reported a lack of such awareness. Awareness was higher among parents of male children (404 (72.5%)) compared to those with female children (283 (63.0%)), with the highest level observed in the western region of India (113 (78.0%)). Additionally, a majority of parents expressed interest in identifying such preventive strategies, as detailed in Table 2. Parental awareness of specific measures to delay the onset of eczema symptoms is further illustrated in Figure 1.

**Table 2:** Interest in identifying strategies to delay atopic dermatitis onset in high-risk children

Response	N (%)
Yes	895 (89.23)
No	108 (10.77)

Familiarity With AD

Of the 1,003 parents surveyed, 862 (86%) responded to the question assessing familiarity with AD. Among these, 698 (69.6%) reported being familiar with the condition. Familiarity was higher among parents of male children (408 (73.3%)) compared to those with female children (290 (65.0%)). Age-wise, the highest familiarity was noted among parents of three-year-old children (167 (78.4%)). Regionally, awareness was most pronounced in North India, where 196 (76.0%) of the respondents indicated familiarity with AD.

Preventive measures to delay the onset of symptoms among children diagnosed with AD

Among the 179 children with AD, a significant majority of parents (144 (80.4%)) reported attempting at least one method to delay the onset of symptoms. Notably, 20 (86%) of parents of one-year-old children indicated efforts to prevent symptom development. The overall usage of preventive treatments and the reasons for using or not using them in children with AD are detailed in Table 3.

**Table 3:** Use of preventive treatments in children with atopic dermatitis before diagnosis to delay symptom onset

Variables	N (%)
Use of preventive treatment (N=179)	
Yes	144 (80.45)
No	33 (18.44)
Not applicable	02 (1.12)
Reasons for using preventive treatment for AD before diagnosis (one or more reasons)	
To protect my child	109 (75.69)
The treatment was easy to incorporate into the daily routine	78 (54.17)
The treatment(s) were easy to access	60 (38.71)
Due to previous family history	53 (36.81)
Reasons for not using preventive treatment for AD before diagnosis (one or more reasons)	
I did not realize there were any	15 (45.45)
I was not aware I needed to use preventive treatment	23 (69.70)
I do not think preventive treatments are worth using	7 (21.21)
These were not available to me due to the cost	6 (18.18)

Preventive treatments in children with a high risk of developing AD

Among the 1,003 respondents, irrespective of having children diagnosed with AD, a substantial majority expressed their willingness to consult healthcare professionals regarding preventive treatments for children at high risk of developing AD. Among respondents who indicated they would consider preventive treatment if their child were at high risk of developing AD, a substantial proportion reported they would seek professional advice. Specifically, 436 (85.83%) parents of male children, 133 (93.66%) parents of one-year-old children, and 366 (90.82%) parents from South India expressed this intent. However, when asked, “If you suspected your child was at high risk of developing AD, what preventive treatment, if any, would you try first?”, only 130 respondents (12.96%) identified moisturizer use as their preferred initial intervention. The parental choices of preventive treatments, if they are ever used, and the reasons for using or not using them in children at high risk of developing AD are detailed in Table 4.

**Table 4:** Preventive actions taken if the child is suspected to be at high risk of developing atopic dermatitis

Variables	N (%)
Preventive treatments (N=1003)	
Consult a healthcare professional	770 (76.77)
Use a moisturizer	130 (12.96)
Not sure/undecided	51 (5.08)
Not take any specific action	46 (4.59)
Others	6 (0.60%)
Reasons for using preventive treatments in high-risk children (one or more reasons) (N=906)	
To protect my child	790 (87.20)
These seem easy to access/use	317 (34.99)
It is not worth the risk of not using the preventive treatment	209 (23.07)
Reasons for not using preventive treatments in high-risk children (one or more reasons) (N=97)	
Not sure what this would require	49 (50.52)
I do not think preventive treatments are worth using	37 (38.14)
The potential cost of using these	30 (30.93)

Consultation for eczema/AD

Most parents (555 (55.4%)) indicated that they would first consult a dermatologist for managing eczema or AD in their children, reflecting a preference for seeking clinical advice primarily for diagnosis and treatment. Details on consultation preferences are provided in Table 5.

**Table 5:** First consultation preferences for eczema/atopic dermatitis

Preferred consultations	N (%)
Dermatologist	554 (55.23)
Pediatrician	274 (27.32)
Midwife	47 (4.69)
Nurse	65 (6.48)
Not sure	60 (5.98)
Others	3 (0.30)

Moisturizer use in children at high risk of developing AD

**Figure 2:**
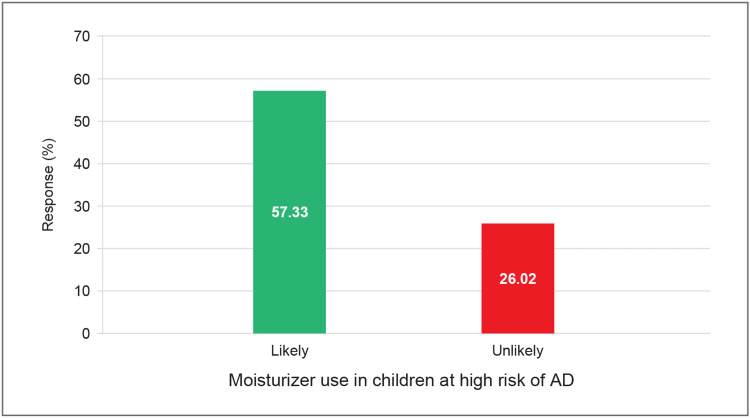
Use of moisturizers before symptom onset in children at high risk of developing atopic dermatitis

Among all the respondents, 575 (57.3%) parents said they would be likely to use moisturizers if they perceived their child to be at high risk of developing AD, as depicted in Figure 2.

The use of moisturizers as a preventive measure for AD before symptom onset was more common among specific groups. Parents of female children reported a 256 (57.4%) likelihood of using moisturizers, while 146 (65.47%) of parents with 4-year-old children and 159 (61.87%) of those from North India also indicated a higher propensity to employ this preventive strategy.

Symptom flare-ups in children with AD

Among the 179 (17.85%) children with AD, most parents (119 (66.4%)) treated their child's symptoms within one week of noticing a flare. Most of the children treated within this timeframe were nearly four years old, representing 27 (72.97%) of the respondents. The overall time taken to treat the flare-up of symptoms in children with AD is given in Table 6.

**Table 6:** Duration of treatment for symptom flare-ups in children

Response	N (%)
I treated my child's symptoms within one week	119 (66.48)
I waited between one week and one month to treat my child's symptoms	44 (24.58)
I waited more than one month to treat my child's symptoms	16 (8.94)
I did not treat my child's symptoms during a flare-up	(0)

Factors influencing the topical treatment for children with AD

Parents identified safety as the primary consideration when selecting topical treatments for children with AD (829 (82.65%)). The factors influencing treatment selection are detailed in Table 7.

**Table 7:** Factors considered when selecting a topical treatment for children with atopic dermatitis

Parameters			Factor	N (%)
Safety		Important		829 (82.65)
		Unimportant		103 (10.27)
Effectiveness		Important		793 (79.06)
		Unimportant		117 (11.67)
Ease of use/application		Important		734 (73.18)
		Unimportant		111 (11.07)
Cost		Important		531 (52.94)
		Unimportant		208 (20.74)
Dermatologist-recommended		Important		795 (79.26)
		Unimportant		119 (11.86)

**Figure 3:**
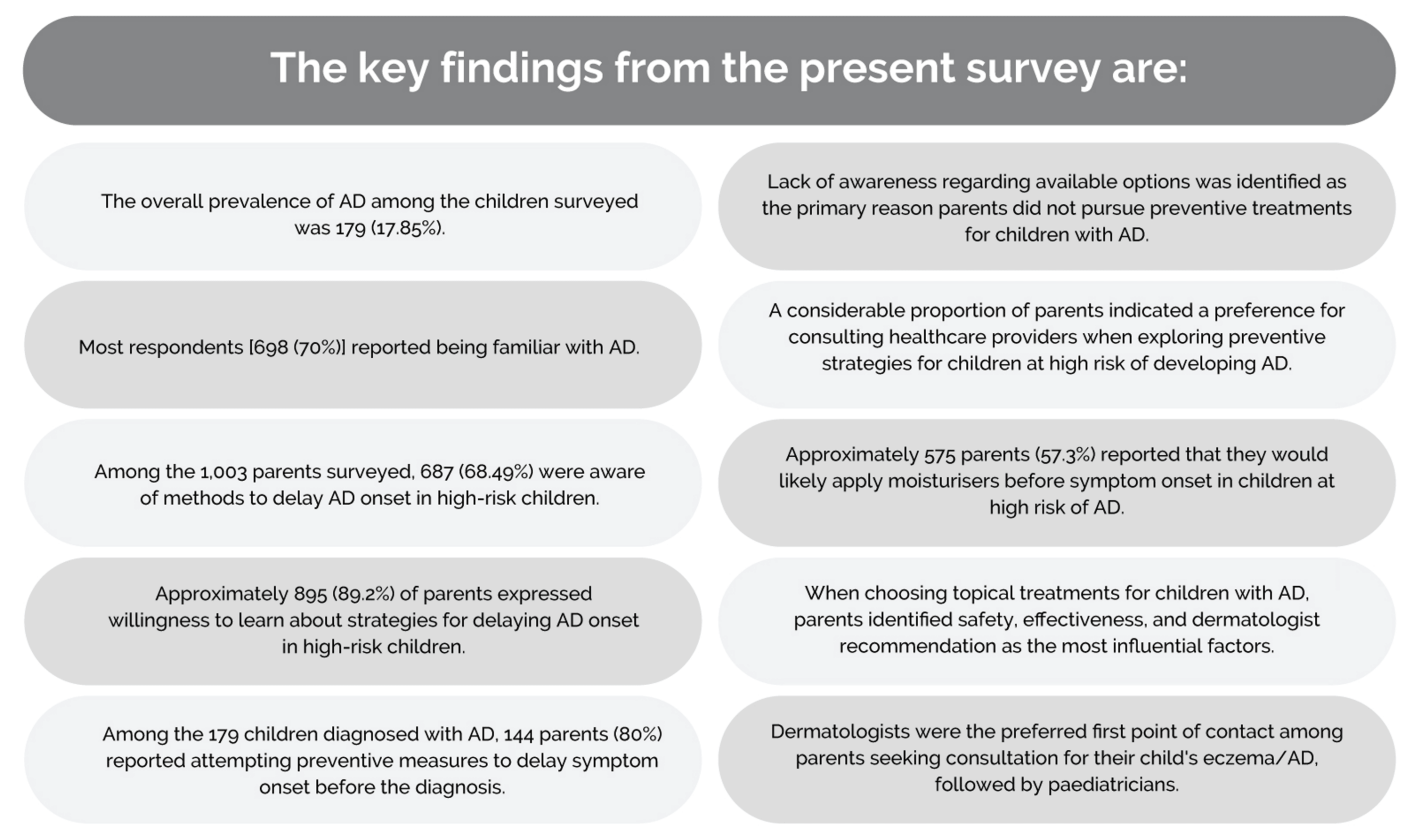
Key findings from the survey AD: atopic dermatitis

**Figure 4:**
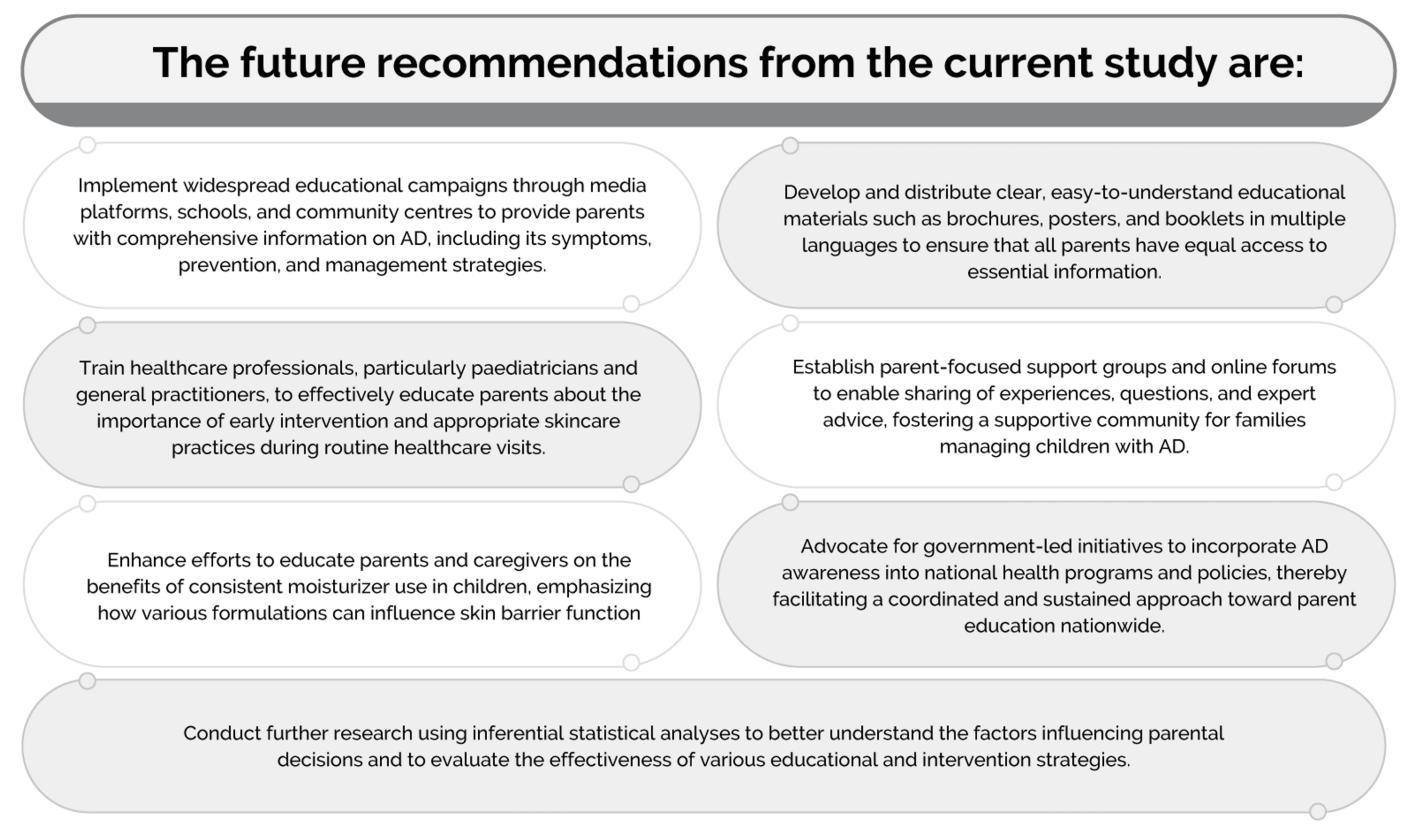
Future recommendations from the current survey AD: atopic dermatitis

The key findings from the present survey and the future recommendations derived from this study are presented in Figures 3, 4, respectively.

## Discussion

The growing prevalence of AD in children has made it a significant public health concern [20]. In the global scenario, the current prevalence of AD in children is 10%-30% in high-income and some low-income countries, signifying a two-to-three-fold rise during the last few decades [21]. Although limited, available population-based studies suggest considerable regional variation in the epidemiology of AD across India. For instance, a study from Eastern India reported a low prevalence of 0.55%, while one from Northern India documented a prevalence rate of 29.9% [22]. These discrepancies may be attributed to India's diverse climatic conditions, which can influence disease patterns. Consistent with this, our survey found that 179 (17.85%) children were diagnosed with AD, with notably higher proportions reported in certain regions, such as the Northeast, where 11 (20.75%) children were affected. This further highlights the need for large-scale, regionally representative epidemiological studies on pediatric AD in India.

The parental history of atopic diseases is a well-established risk factor for the development of AD in children. Studies indicate that the presence of AD in one parent increases the risk in offspring, with even higher risk observed when both parents are affected [23-26]. In one study involving 130 children aged three months to 1.5 years with AD, a majority had a personal and/or family history of atopy, further emphasizing the strong hereditary component [25]. In the present survey, out of 1003 parents, 655 (65.30%) had a family history of AD.

Food allergies are commonly linked to AD in children under the age of five years. Thus, if a patient with moderate-to-severe AD does not improve with adequate treatment, a food allergy test may be undertaken [27]. In children with AD, 47%-80% report having sleep disturbance [28]. Moreover, up to 83% of children may experience sleep disturbances during eczema flare-ups [29]. In the current survey, parents opined that irritability and sleep disturbances are the most associated factors with AD.

The understanding and attitude of parents toward AD in children are crucial for the effective management of the disease [30]. The attitude of parents and children with AD is shown to have a significant positive correlation [31]. In the survey, 684 (68.4%) parents reported being aware of strategies to delay the onset of AD in high-risk children. Additionally, a large majority (895 (89.23%)) expressed interest in learning more about preventive approaches to delay symptom onset in children at high risk of developing AD.

The present survey showed that 144 (80.45%) parents of children with AD tried using preventive treatments to delay the symptom onset before the diagnosis. Many parents search for information from various sources to help them manage their child's illness. Parents/guardians use various websites or discussion forums to gather information. Unsatisfactory outcomes may result from ignorance about the condition or from misunderstanding it, particularly in the case of parents whose children have AD [30]. Preventive management strategies for children with AD can vary based on several factors, including the severity of the condition, individual triggers, lifestyle factors, and the preferences of the children and their caregivers [32]. In this survey, among the 33 respondents who had not attempted any methods to delay the onset of AD symptoms, 23 (69.70%) cited lack of awareness as the primary reason for not using preventive treatments. Educating parents about their child's AD can help lessen the severity of the disease, enhance the quality of life, improve perceptions of the care received, help the patient build coping mechanisms, and save costs. Moreover, it needs to be a regular part of clinical practice [33].

Although 575 (57.3%) parents said that they were likely to use a moisturizer in case their child was at high risk of developing AD, only 130 (12.96%) used it as a preventive measure. A previous study reported that parents' use of moisturizers for newborns with eczema depends on their understanding of moisturizers' benefits. Approximately 72% of parents knew that moisturizers could help repair the skin barrier, while 50% recognized their role in repairing damaged skin. However, only 7.4% were aware that moisturizers could decrease skin sensitivity, and just 8.6% knew they might reduce recurrent eczema episodes. Additionally, 57.9% (62 out of 107) of parents did not apply moisturizers to their infants with eczema. These findings show that many parents lacked awareness about the benefits of moisturizers, which might have led to insufficient use. Although parents generally understood the importance of moisturizers, they did not use them adequately [14,34]. Notably, “cost” was also cited by over half the parents (52.94%) as an important consideration when selecting topical treatments for children with AD (Table 7). Although the study did not directly evaluate cost-saving outcomes, the parental emphasis on affordability underlines the potential for cost-effective strategies to improve adherence and outcomes, particularly in resource-constrained settings.

Non-pharmacological interventions are important for the better management of AD. Ointments and creams provide a better barrier function than lotions. There is an abundance of evidence to support the efficacy of emollients in the treatment and prevention of pediatric AD. Because of the large body surface area to weight ratio, topical medications are absorbed more readily in infants. This results in a decreased dosage of topical drugs and a greater demand for moisturizers to prevent skin water loss [12]. Appropriately formulated emollients, when free from known irritants such as fragrances, dyes, and harsh preservatives, are generally considered safe and well-tolerated for use on sensitive newborn and infant skin. Emollient therapy has also demonstrated some preventive benefits in populations at risk of developing AD [35]. The regular use of emollients in pediatric patients with AD has been shown to impart steroid-sparing effects, regardless of the severity of the condition [36-38]. These effects are particularly significant in reducing the consumption of high-potency corticosteroids [38]. Emollients are also recommended as an adjuvant to topical anti-inflammatory medications such as corticosteroids and calcineurin inhibitors, with their steroid-sparing properties [38]. A randomized controlled trial on children with moderate-to-severe AD objectively and quantitatively showed the impact of a six-week emollient adjuvant therapy on the decrease in the consumption of topical corticosteroids. It showed that in infants with AD, the high-potency topical corticosteroid use was dramatically decreased by the emollient treatment. The study's findings on the corticosteroid-sparing effect of emollients could be explained by their ability to strengthen the skin barrier, reducing the penetration of allergens and irritants that trigger skin inflammation. Additionally, the emollient used in the study had active oat extracts, which may have had anti-inflammatory qualities in alleviating skin inflammatory conditions like AD [38]. Historically, colloidal oatmeal has been used in the treatment of various dermatological conditions due to its anti-inflammatory and soothing properties. Over-the-counter 1% oatmeal-based creams are as safe and effective as prescription barrier creams for the symptomatic management of mild-to-moderate AD in children [39]. In our survey, 575 (57.3%) parents indicated they were likely to use a moisturizer proactively in children at high risk of developing AD. Among the key factors influencing their choice of topical products were safety, effectiveness, and dermatologist recommendations.

Reljić et al. reported that parents most frequently obtained information about AD from treating dermatologists (63 (90%)) and the Internet (56 (80%)) [30]. Our findings also confirmed dermatologists to be the first choice of parents for consultations about their children with eczema/AD.

Study limitations and future directions

While this survey included participants from various regions across India, the sample size of 1,003 might not fully represent the entire population. Additionally, as the study relied on self-reported data, there was a potential for recall bias and subjective interpretation of AD-related knowledge and management practices. The study primarily employed descriptive statistical analysis, and the lack of inferential statistical methods limited the ability to draw broader conclusions about associations between parental knowledge, attitudes, and AD management practices. Additionally, the survey item assessing the family history of atopic conditions did not specifically restrict responses to first-degree relatives. As such, the reported prevalence of family history may reflect a broader familial interpretation by the respondents, which should be considered when interpreting the findings.

Future research should incorporate larger, more diverse samples and inferential statistical analyses to explore potential predictors of parental awareness, access to care, and treatment adherence. Integrating clinical validation alongside parental reporting could provide a more comprehensive assessment of AD prevalence and management strategies in India. Furthermore, parental awareness of the role and benefits of moisturizers in managing children with AD should be enhanced. Future awareness programs should focus on educating parents about moisturizer ingredients, their impact on the skin barrier, and their role in AD management, as these measures may help improve long-term treatment adherence in children. Factors such as forgetfulness, sensory discomfort, or difficulty integrating moisturization into daily routines may affect compliance. Therefore, targeted awareness programs emphasizing early and consistent moisturizer use are essential for optimizing AD management.

## Conclusions

This study provides insights into parental KAP regarding AD in India, highlighting gaps in awareness and early intervention strategies. While the prevalence of AD among the surveyed pediatric population aligns with existing literature, a significant proportion of parents remain unaware of effective management options, particularly the role of moisturizers in maintaining skin barrier function and reducing flare-ups. The findings highlight the need for structured educational initiatives to enhance parental understanding of AD management, particularly regarding early intervention and appropriate skincare practices. In this survey, most parents expressed interest in learning about strategies to delay AD onset in high-risk children. The data suggest that targeted awareness programs and accessible healthcare policies could bridge these gaps, leading to better disease management and improved quality of life for children with AD. While some aspects of the conclusion suggest opportunities for future interventions, these are intended as forward-looking recommendations based on survey insights rather than outcomes derived from hypothesis-driven testing. Further research with analytical statistical methods and clinical validation is essential to establish causal relationships and assess the effectiveness of specific interventions. Collaborative efforts among healthcare professionals and policymakers are critical for developing evidence-based strategies to optimize AD care in India.

## Appendices

Appendix A 

**Table 8:** Survey Instrument for the Pediatric Sensitive Skin Study

Questions										
Q1. How familiar, if at all, are you with the condition atopic dermatitis?										
Q2. Do you or anyone in your immediate family have atopic dermatitis, or other allergic conditions such as, food allergies, rhinitis* or asthma? Select all that apply										
Q3. Do you have a child with atopic dermatitis?										
Q4. Before your child was diagnosed with atopic dermatitis, did you try any preventative treatments to delay the onset of symptoms?										
Q4a. Why did you try preventative treatments for atopic dermatitis? Select all that apply										
Q4b. Why did you not try any preventative treatments for atopic dermatitis? Select all that apply										
Q5. Which of the following, if any, do you think is related to atopic dermatitis? Select all that apply										
Q6. Which of the following conditions, if any, if found in your family history, do you think can affect the likelihood of having atopic dermatitis? Select all that apply										
Q7. To what extent, if at all, are you aware there are ways to delay the onset of atopic dermatitis in high-risk children?										
Q7a. Would you be interested in finding out more about the ways to delay the onset of atopic dermatitis in high-risk children?										
Q8. If you suspected your child was at high-risk of developing atopic dermatitis, what preventative treatment, if any, would you do first:										
Q8a. Why would you use this preventative treatment? Select all that apply										
Q8b. Why would you not use a preventative treatment? Select all that apply										
Q9. Which of the following would you do first:										
Q10. If you suspected that your child was at a high risk of developing atopic dermatitis, how likely or unlikely would you be to use a moisturiser before the symptoms appear?										
Q11. When your child experiences a flare (worsening of symptoms), how long did you take to treat the symptoms after the symptoms appear?										
Q12. Did you know there were ways you could delay the onset of eczema symptoms?										
Q13. Which of the following factors do you MOST associate with atopic dermatitis in children?										
Q14. Please rate the following factors in terms of their importance to you when selecting a topical treatment for your child. Use a scale of 1 to 5, with 5 indicating the highest importance and 1 indicating the lowest importance:										
Q15. Who, if anyone, would you/did you consult first for eczema/atopic dermatitis?										
Censuswide abides by and employs members of the Market Research Society which is based on the ESOMAR principles.										
